# Post-exposure treatment of non-human primates lethally infected with Ebola virus with EBOTAb, a purified ovine IgG product

**DOI:** 10.1038/s41598-017-03910-7

**Published:** 2017-06-22

**Authors:** Stuart D. Dowall, Frédéric Jacquot, John Landon, Emma Rayner, Graham Hall, Caroline Carbonnelle, Hervé Raoul, Delphine Pannetier, Ian Cameron, Ruth Coxon, Ibrahim Al Abdulla, Roger Hewson, Miles W. Carroll

**Affiliations:** 1 National Infection Service, Public Health England, Porton Down, Salisbury, Wiltshire SP4 0JG UK; 2 Laboratoire P4, INSERM Jean Merieux, 21 Avenue Tony Garnier, Lyon, France; 3 MicroPharm Ltd, Station Road, Newcastle Emlyn Dyfed, SA38 9BY UK

## Abstract

Despite sporadic outbreaks of Ebola virus (EBOV) over the last 4 decades and the recent public health emergency in West Africa, there are still no approved vaccines or therapeutics for the treatment of acute EBOV disease (EVD). In response to the 2014 outbreak, an ovine immunoglobulin therapy was developed, termed EBOTAb. After promising results in the guinea pig model of EBOV infection, EBOTAb was tested in the cynomolgus macaque non-human primate model of lethal EBOV infection. To ensure stringent therapeutic testing conditions to replicate likely clinical usage, EBOTAb was first delivered 1, 2 or 3 days post-challenge with a lethal dose of EBOV. Results showed a protective effect of EBOTAb given post-exposurally, with survival rates decreasing with increasing time after challenge. Viremia results demonstrated that EBOTAb resulted in a decreased circulation of EBOV in the bloodstream. Additionally, assay of liver enzymes and histology analysis of local tissues identified differences between EBOTAb-treated and untreated groups. The results presented demonstrate that EBOTAb conferred protection against EBOV when given post-exposure and should be explored and developed further as a potential intervention strategy for future outbreaks, which are likely to occur.

## Introduction

Whilst Ebola virus (EBOV) was first identified in 1976^1^, there are still no licensed therapeutics or vaccines available to treat or protect against infections; although several therapies^2^ and vaccines^3^ are progressing through clinical trials. With the increasing ease and speed of global travel, and it’s potential to spread via the aerosol route^4^, EBOV is a public health threat^5^ due to the high mortality rate and lack of approved interventions. The largest outbreak of EBOV occurred in Western Africa and was first recognised in March 2014^6^, resulting in more deaths than all previous outbreaks combined. This large outbreak catalysed increased efforts to identify and evaluate potential prophylactic and therapeutic options.

Whilst developments of vaccines have shown great promise against EBOV^7–9^, they may not offer a full solution due to the cost associated with vaccinating the population of a large region in order to confer an effective level and distribution of immunity. Therefore, a post-exposure treatment for EBOV is urgently required. Several options have been assessed that have demonstrated protective effects in non-human primate (NHP) models of EBOV including hyperimmune equine IgG^10^, recombinant nematode anticoagulant protein C2^11^, recombinant human activated protein C^12^, recombinant vesicular stomatitis virus vectors^13^, small interfering RNA^14^ and phosphorodiamidate morpholino oligomers^15, 16^. Treatments in the aforementioned studies were typically started within 24 hours after EBOV challenge and the majority of treatments were administered within 1 hour post-challenge.

Antibody treatment against EBOV has a chequered history, with several reports indicating that passive immunotherapy in NHPs failed to confer protection^10, 17–19^. However, more recently antibodies have received extra attention with the development of monoclonal antibody treatments demonstrating efficacy^20–24^ and the humoral component of the immune system being necessary for vaccine-induced protection^25, 26^ against lethal EBOV challenge in NHP studies.

In response to the 2014 West Africa EBOV outbreak, an ovine immunoglobulin preparation was rapidly developed, termed EBOTAb, which demonstrated neutralisation activity and exhibited promising results in the EBOV guinea pig model^27, 28^. Due to the outbred guinea pig model of EBOV infection showing coagulopathy^29^, this model is regarded as a more authentic model of human disease than mice or inbred guinea pig models and an important animal system^30^. However, the finding that a potent humanised neutralising antibody, KZ52, protected guinea pigs^31, 32^ but not NHPs^19^, the need to assess anti-EBOV therapies in NHP’s is paramount. NHPs are the accepted current gold standard^33^, and bear similarities to the pathogenesis of human infection^34–38^. Therefore, the next logical step for the preclinical testing of EBOTAb to demonstrate its potential utility for clinical development was assessment in a NHP model of EBOV infection. To ensure that EBOTAb was tested stringently, dosing was initiated at either 1, 2 or 3 days post-challenge with a lethal dose of EBOV.

## Results

### EBOTAb confers therapeutic effects against lethal EBOV infection when treatment is delayed up to 3 days post-challenge

**Figure 1:**
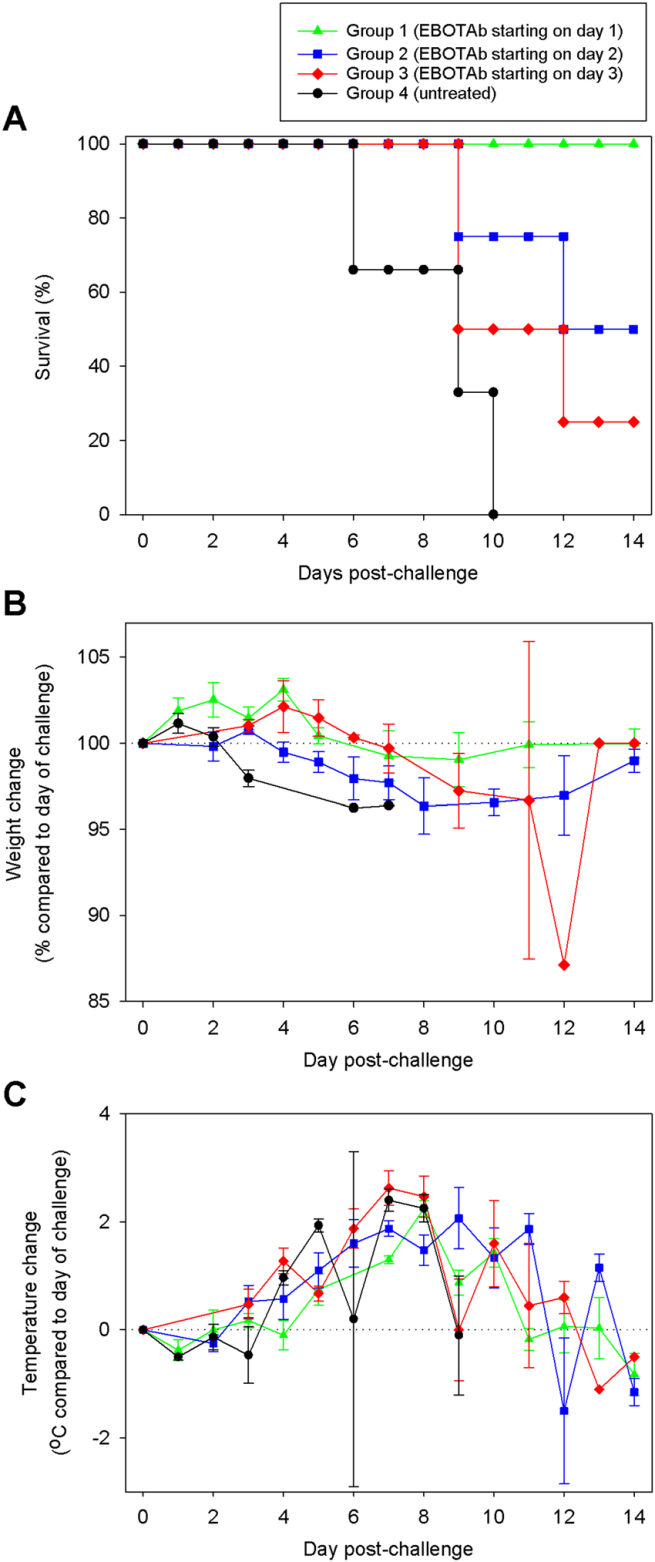
Survival and clinical parameters of NHPs after treatment with EBOTAb starting at 1, 2 and 3 days post-challenge. (**A**) Kaplan-Meier survival plot of animals challenged with 10^3^ pfu EBOV before treatment with EBOTAb on day 1, 2 and 3 post-challenge. (**B**) Weight difference of animals in the study compared to weights recorded on the day of challenge. Mean weight changes are plotted with error bars denoting standard error. (**C**) Temperature difference of animals in the study compared to the temperature recorded on the day of challenge. Mean temperature changes are plotted with error bars denoting standard error.

To assess the therapeutic potential of EBOTAb, treatment was initiated at 1, 2 or 3 days post-challenge. Untreated animals met humane endpoints by day 10 post-challenge. An increase in survival was observed after treatment with EBOTAb, with survival rates of 100% (4 of 4), 50% (2 of 4) and 25% (1 of 4) for the treatment starting at 1, 2 or 3 days post-challenge, respectively (Fig. 1A). The increase in survival was statistically significant in the day 1 group with the significance decreasing as the time post-challenge increased (P = 0.010, P = 0.062 and P = 0.1848 for treatment starting on days 1, 2 and 3, respectively, Log-Rank survival analysis). During the course of the study, body weight and temperature were also routinely measured. Untreated animals lost weight much earlier than the EBOTAb-treated groups (Fig. 1B). All EBOTAb-treated animals lost weight during the course of the study. The weight of those which survived to day 14 post-challenge had increased indicating a recovery from EBOV infection. No marked differences in body temperature between untreated and EBOTAb-treated groups were observed, with all animals showing a rise in temperature during the course of the study (Fig. 1C).

### Treatment with EBOTAb reduces plasma viremia levels

**Figure 2:**
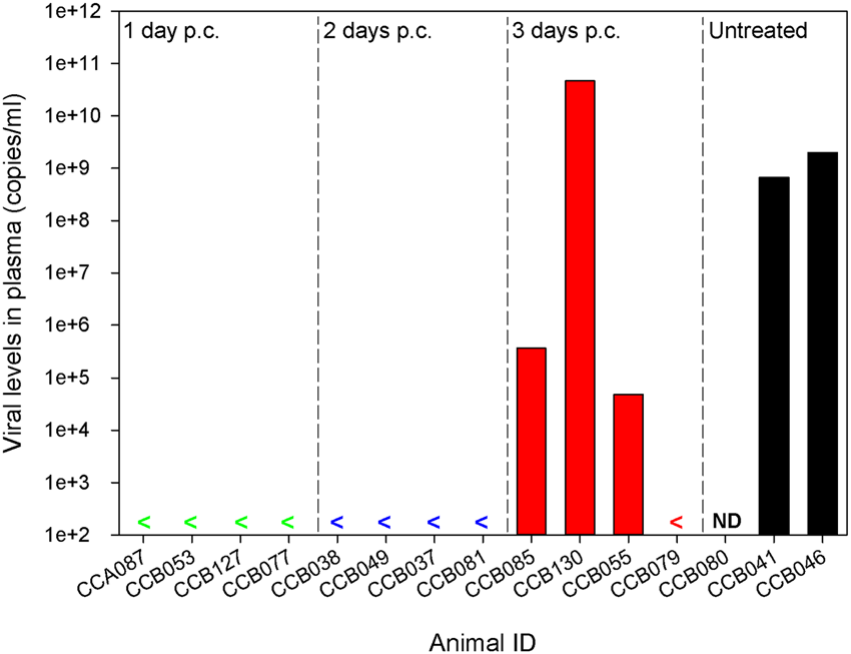
Viral RNA levels in the plasma of EBOV-infected animals treated with EBOTAb at 1, 2 and 3 days post-challenge. Seven days post-challenge with EBOV, a blood sample was collected from all animals for the assessment of viremia via RT-PCR. Data was calculated to give a readout of genome copies per ml. Results are shown from individual animals. ND, not done as this animal had met a humane endpoint prior to the date of blood withdrawal.

At day 7 post-challenge, a blood sample was collected from the animals to assess the levels of EBOV RNA in the plasma. High levels of viral RNA was detected in untreated animals, whereas in those that received EBOTAb at 1 or 2 days post-challenge were not viremic and viremia was detected in three of four animals whose treatment was delayed to 3 days post-challenge (Fig. 2). Of the 3 animals showing viremia in the group where treatment was delayed to day 3 post-challenge, 2 were at >3-log lower levels compared to those observed in the untreated group indicating a significant reduction in circulating viral load.

### Viral load in the liver and spleen of EBOTAb-treated animals is dependent on time when treatment was first delivered

**Figure 3:**
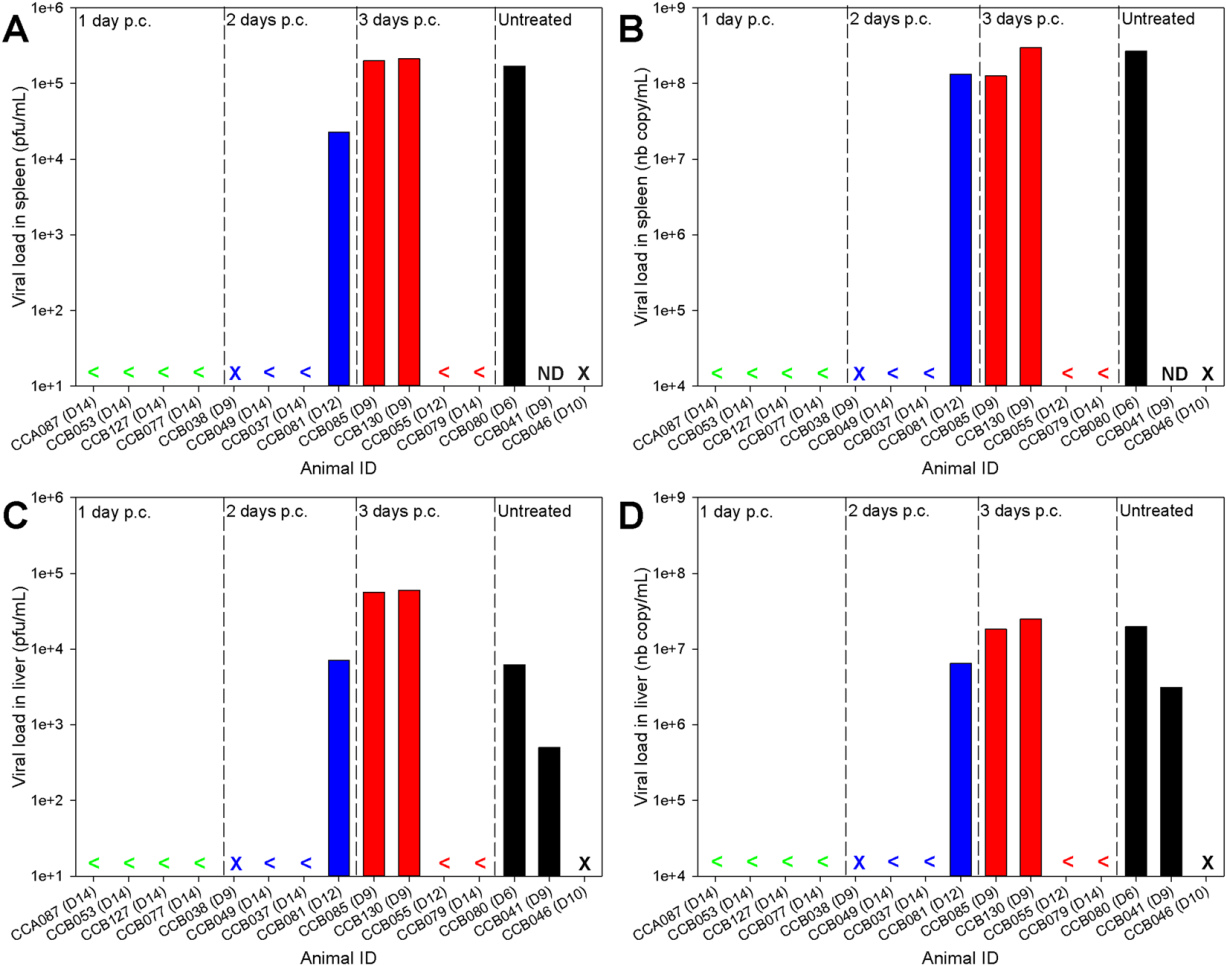
Viral RNA levels in the spleen and liver of EBOV-infected animals treated with EBOTAb at 1, 2 and 3 days post-challenge. When animals reached humane endpoints or at the scheduled end of the study, the levels of EBOV were measured by RT-PCR and plaque assay. (**A**) Spleen, plaque assay. (**B**) Spleen, RT-PCR assay. (**C**) Liver, plaque assay. (**D**) Liver, RT-PCR assay. Plaque assay results are displayed as plaque-forming unit per gram of tissue. RT-PCR results are displayed as genome copies per gram of tissue. <denotes no detectable signal, ND denotes sample not assessed due to sample not being collected at necropsy and X indicates animal had died before reaching humane endpoints so samples were not collected.

When animals met humane endpoints or at the end of the study (14 days post-challenge), samples of liver and spleen were collected for analysis of virus levels by RT-PCR and plaque assay, with both assays demonstrating similar results (Fig. 3). Viral RNA levels in the spleen (Fig. 3A and B) were higher than those observed in the liver (Fig. 3C and D). The results demonstrated similar levels of viral RNA in the animals which met humane endpoints before the end of the study, apart from animal CCB055 that had no EBOV RNA detectable despite meeting humane endpoints on day 12 post-challenge. All animals which survived to the scheduled end of the study, 14 days post-challenge, had no viral RNA detected in either the liver or spleen indicating clearance of the virus to undetectable levels.

### Biochemical markers indicated reduced liver damage in EBOTAb-treated animals compared to untreated controls

**Figure 4:**
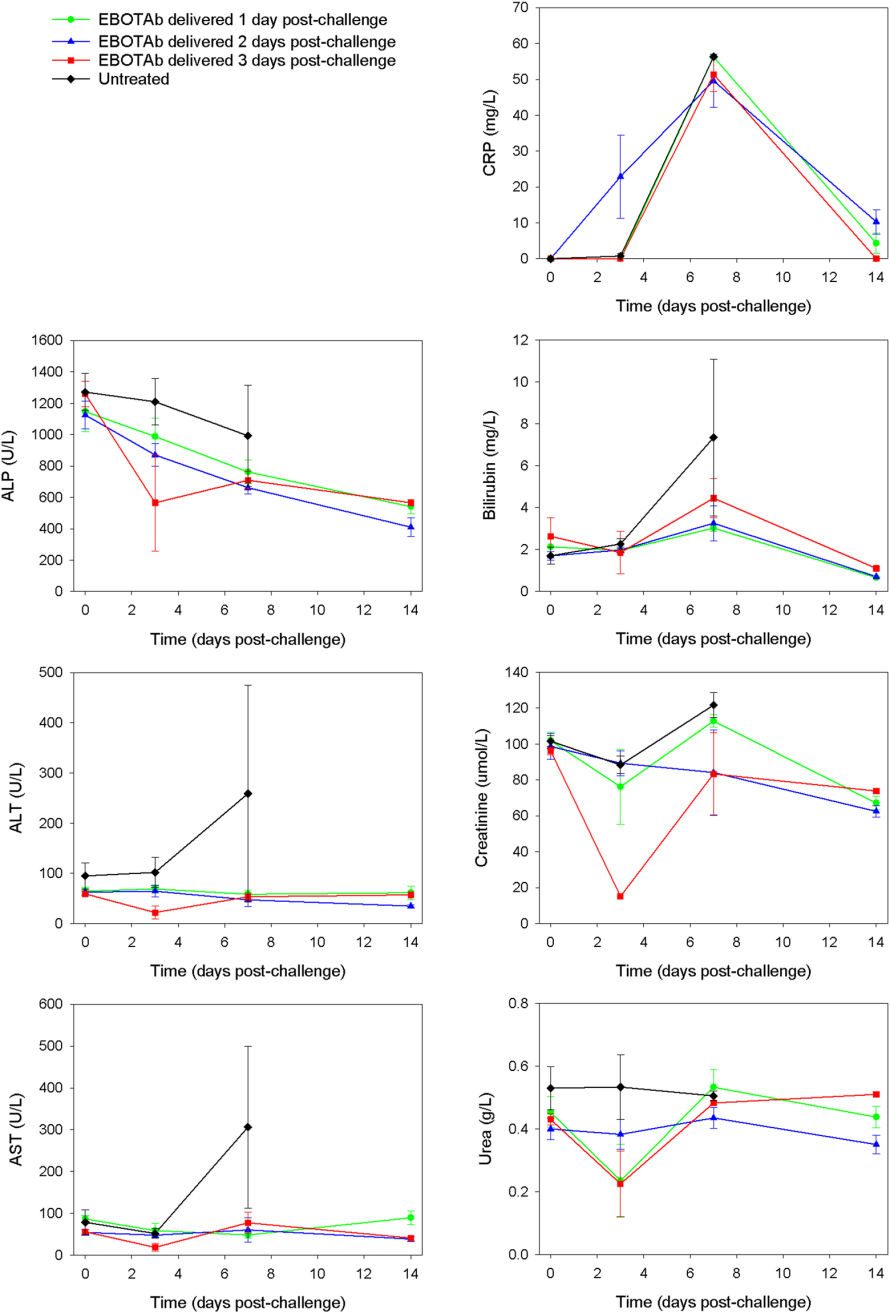
Biochemical analysis for markers of inflammation, liver function and kidney function in animals treated with EBOTAb at 1, 2 and 3 days post-challenge. Blood samples were collected from animals on the day of challenge (day 0) and at 3, 7 and 14 days post-challenge from animals still surviving at these timepoints. Mean results are plotted with error bars denoting standard error. *Denotes statistical difference between treated group and untreated group (P = 0.0518, Mann-Whitney statistical test).

During the course of the study, blood was collected for biochemistry analysis to assess concentrations of C-reactive protein (CRP), alkaline phosphatase (ALP), alanine transaminase (ALT), aspartate aminotransferase (ASP), bilirubin, creatine and urea in EBOTAb-treated animals compared to untreated macaques (Fig. 4). Concentrations of CRP were similar across all groups, with a noticeable peak on day 7 post-challenge. For the liver enzymes, there were no discernible differences in ALP concentrations but with ALT, AST and bilirubin the untreated animals showed an increase in concentrations at the day 7 timepoint. For the kidney proteins, creatinine and urea, no differences between EBOTAb-treated and untreated animals were observed.

### No difference in haematological markers were observed in EBOTAb-treated animals compared to untreated controls

**Figure 5:**
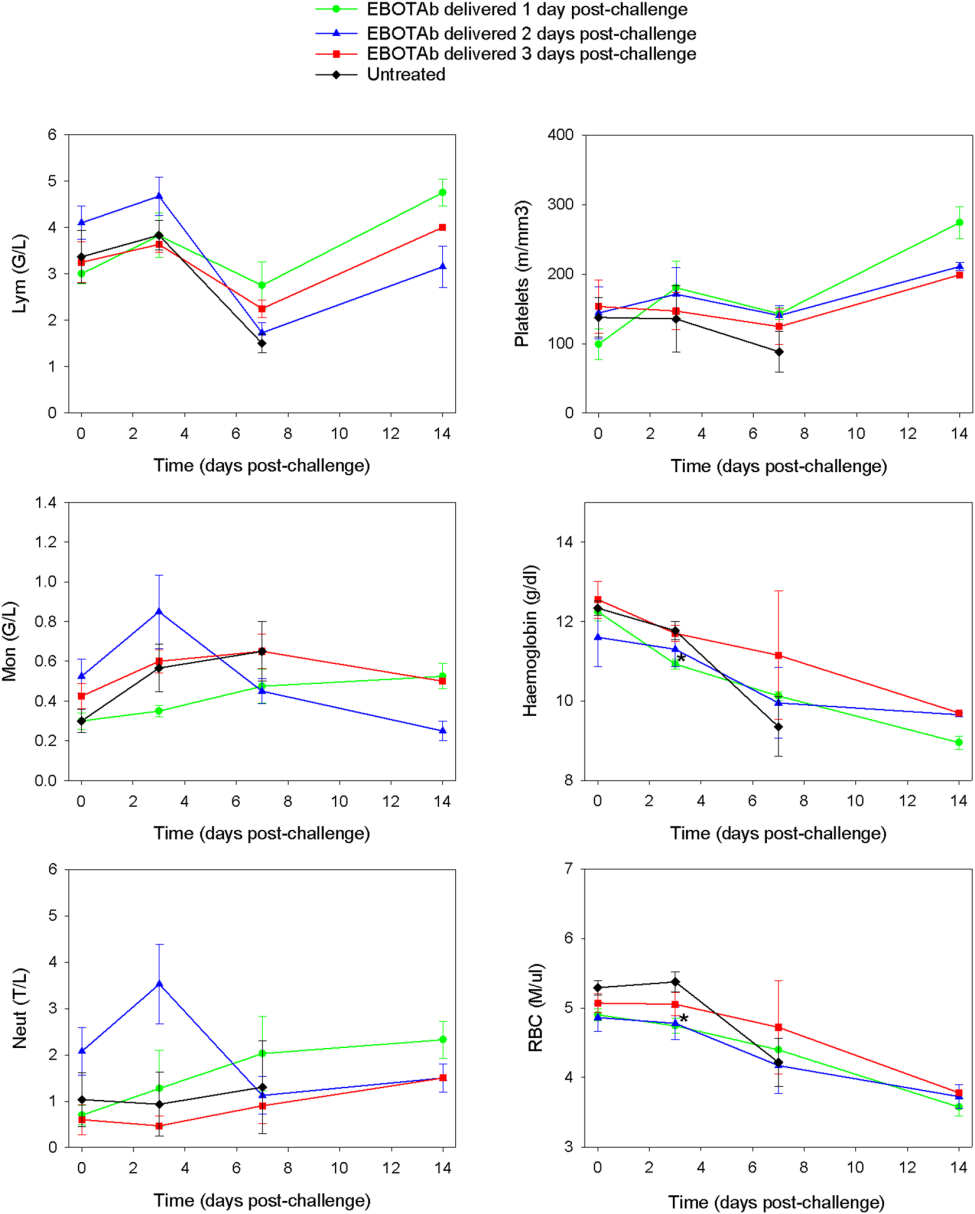
Haematological analysis in animals treated with EBOTAb at 1, 2 and 3 days post-challenge. Blood samples were collected from all animals on the day of challenge (day 0) and at 3, 7 and 14 days post-challenge from animals still surviving at these timepoints. Mean results are plotted with error bars denoting standard error. Abbreviations: Lym, lymphocytes; Mon, monocytes; Neut, neutrophils; and RBC, red blood cells. *Denotes statistical difference between treated group and untreated group (P = 0.0518, Mann-Whitney statistical test).

In parallel to the biochemistry analysis, blood was also collected for quantitation of haematological subsets, including lymphocytes, monocytes, neutrophils, platelets, red blood cells and haemoglobin concentration. No discernible differences were observed between those animals which received EBOTAb post-challenge compared to untreated controls (Fig. 5).

### Histological analysis of tissue samples demonstrated that EBOTAb treatment reduced tissue damage severity

When animals met humane endpoints or at the scheduled end of the study, samples of liver, spleen, inguinal lymph node and lung were immersed in 10% normal buffered formalin, processed for histological analysis and stained with haematoxylin and eosin (HE) to assess cellular architecture.

**Figure 6:**
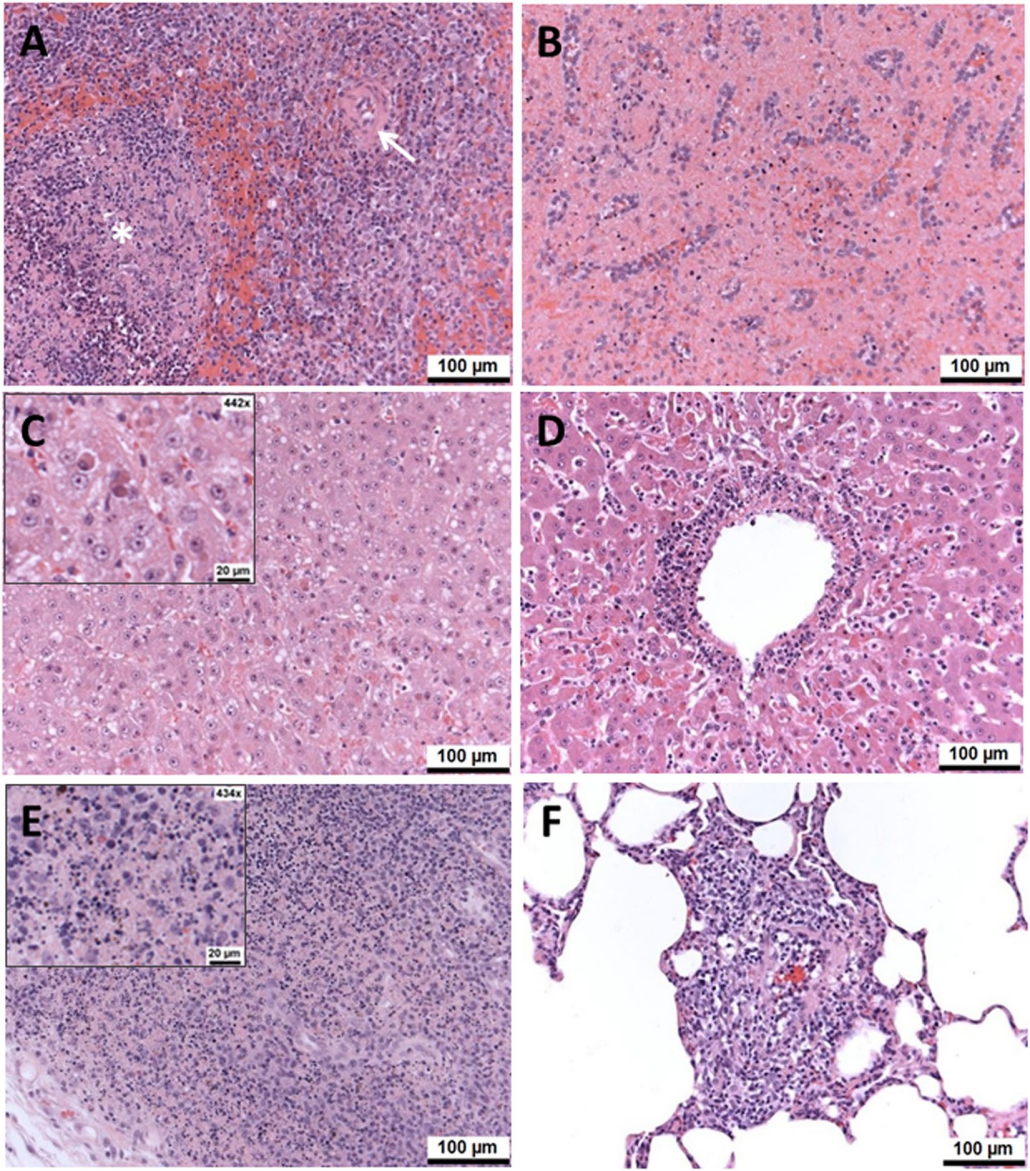
Histological changes in spleen, liver, inguinal lymph node and lungs of EBOV-challenge animals and treated with EBOTAb at 1, 2 and 3 days post-challenge. (**A**) Spleen, untreated (CCB080), day 14 post-challenge. Area of white pulp with severe lymphocyte depletion from a follicle shaped area (asterisk). Arteriole is identified by the arrow. (**B**) Spleen, EBOTAb-treated on day 3 (CCB130), day 9 post-challenge. Scattered nuclear debris within the red pulp. (**C**) Liver, untreated (CCB041), day 9 post-challenge. Scattered, single cell, hepatocellular necrosis. (**D**) Liver, EBOTAb-treated on day 2 (CCB081), day 12 post-challenge. Marked degeneration of a vessel wall with inflammatory cell infiltration. (**E**) Inguinal lymph node, EBOTAb-treated on day 2 (CCB081), day 12 post-challenge. Higher power image showing prominent lymphocyte apoptosis, with nuclear fragmentation (apoptotic bodies) and scattered macrophages in the superficial cortex. (**F**) Lung, EBOTAb-treated on day 2 (CCB037), day 14 post-challenge. Perivascular infiltration by mixed inflammatory cells.

In the spleen, acute lesions associated with EBOV infection were observed both in the red and white pulp of animals which met humane endpoints. In the white pulp, lymphocyte loss was often most prominent from lymphoid follicles. Surrounding peripheral lymphocytes, including those around the arteriole, peri-arteriolar lymphoid sheaths, were less severely affected (Fig. 6A). This change was variably accompanied by foci of congestion and/or haemorrhage. Lymphocyte destruction was characterised by nuclear fragmentation, often accompanied by macrophage infiltration (Fig. 6A). In the red pulp, diffuse, single cell lymphocyte apoptosis was observed (Fig. 6B). Congestion was noted variably.

Necrosis was noted in the liver of animals that progressed to severe disease and met humane endpoints. Focal areas were noted of cellular degeneration, nuclear pyknosis, kayorrhexis and variable polymorphonuclear leukocyte (PMN) infiltration, scattered randomly within the parenchyma. In addition, diffusely scattered, single cell, hepatocellular necrosis was observed comprising cytoplasmic hyper-esoinophilia, and nuclear degradation and loss (Fig. 6C). Occasionally, necrosis and inflammation of blood vessel walls was seen (Fig. 6D).

In inguinal lymph nodes, lymphocyte paucity together with a variable presence of macrophage-like cells was observed widely. In addition, lymphocyte apoptosis was associated variably with this change, comprising apoptotic bodies scattered diffusely within the cortex, as well as focally within follicles (Fig. 6E).

In lung, it was not possible to evaluate alveolar walls accurately due to the variably collapsed nature of the parenchyma. Therefore, only prominent changes were scored. Patchy infiltration of alveolar walls and perivascular areas by mixed inflammatory cells were observed in some animals (Fig. 6F).

In kidney, microscopic lesions were observed in some animals; these were considered as incidental, background changes and not obviously associated with infection with EBOV. Likewise, microscopic changes were not observed in the heart from any animal.

The presence and severity of the histological changes were scored and tabulated (Table 1). The results demonstrated that EBOTAb-treated animals which survived until the scheduled end of the study had fewer changes compared to animals which met humane clinical endpoints.

**Table 1:** Severity of histological findings in EBOV-challenge animals treated with EBOTAb starting at days 1, 2 or 3 post-challenge.

Organ	Description	Treatment groups Animal ID’s (day post-challenge samples collected)												
		EBOTAb 1 day post-challenge				EBOTAb 2 days post-challenge			EBOTAb 3 days post-challenge				Untreated	
		CCA087 (14)	CCB053 (14)	CCB077 (14)	CCB127 (14)	CCB037 (14)	CCB049 (14)	CCB081 (12)	CCB055 (12)	CCB079 (14)	CCB085 (9)	CCB130 (9)	CCB041 (9)	CCB080 (6)
Spleen	Scattered, single cell destruction in red pulp	WNL	WNL	WNL	WNL	WNL	WNL	Mkd	WNL	WNL	Mod	Mkd	Mod	Mkd
	Scattered, single cell destruction in white pulp	WNL	WNL	WNL	WNL	WNL	WNL	Mkd	WNL	WNL	Mod	Mkd	Mild	Mkd
	Lymphocyte loss in white pulp	WNL	WNL	WNL	WNL	WNL	WNL	Mkd	WNL	WNL	Mod	Mkd	Mild	Mod
Liver	Focal necrosis	Min	WNL	WNL	WNL	WNL	WNL	Mkd	WNL	WNL	Mod	Mkd	Mild	Mod
	Single cell, hepatocellular necrosis	WNL	WNL	WNL	WNL	WNL	WNL	Mod	WNL	WNL	Mod	Mod	Mod	Mod
Inguinal lymph nodes	Lymphocyte apoptosis	WNL	WNL	WNL	WNL	WNL	Min	Mkd	Mild	WNL	Mild	Mild	Mild	Mkd
	Lymphocyte paucity+/− macrophage prominence	WNL	WNL	WNL	WNL	WNL	WNL	Mkd	Mkd	WNL	Mod	Mild	Mild	Mod
	Focal necrosis – medulla	WNL	WNL	WNL	WNL	WNL	Min	WNL	WNL	WNL	WNL	WNL	WNL	WNL
Lung	Infiltration by mixed inflammatory cells	WNL	WNL	WNL	WNL	Mild	WNL	WNL	WNL	WNL	Mild	WNL	WNL	WNL

Abbreviations: WNL, within normal limits; Min, minimal; Mod, moderate; Mkd, marked.

### Staining of EBOV antigen showed differences in EBOTAb-treated animals which survived to day 14 compared to untreated animals and those which met humane endpoints

Formalin-fixed sections were stained with an EBOV-specific antibody to identify viral antigen within tissues by immunohistochemical (IHC) analysis (Table 2).

**Table 2:** Presence of viral antigen staining in tissues of animals treated with EBOTAb starting at 1, 2 or 3 days post-challenge.

Treatment group	Animal ID (days from challenge to euthanasia)	Presence of viral antigen					
		Spleen	Liver	Inguinal lymph nodes	Kidneys	Lung	Heart
EBOTAb administered 1 day post-challenge	CCA087 (14)	+	+	+	[+]	+	[+]
	CCB053 (14)	−	−	+	−	[+]	[+]
	CCB077 (14)	+	−	+	[+]	[+]	−
	CCB127 (14)	+	−	+	−	[+]	−
EBOTAb administered 2 days post−challenge	CCB037 (14)	+	−	−	−	[+]	−
	CCB049 (14)	+	+	+	+	+	+
	CCB081 (12)	+++	++	+++	++	++	+
EBOTAb administered 3 days post−challenge	CCB055 (12)	−	−	+	−	[+]	−
	CCB079 (14)	+	−	−	−	[+]	−
	CCB085 (9)	+++	+++	++	+	++	+
	CCB130 (9)	+++	+++	+++	++	+++	++
Untreated	CCB041 (9)	++	++	++	++	++	−
	CCB080 (6)	+++	++	+++	+++	++	++

Legend: +, positively stained areas observed occasionally; ++, positively stained areas observed frequently; +++, numerous, positively stained areas observed; [+], pale, often homogenous, staining of plasma in some blood vessel lumina.

**Figure 7:**
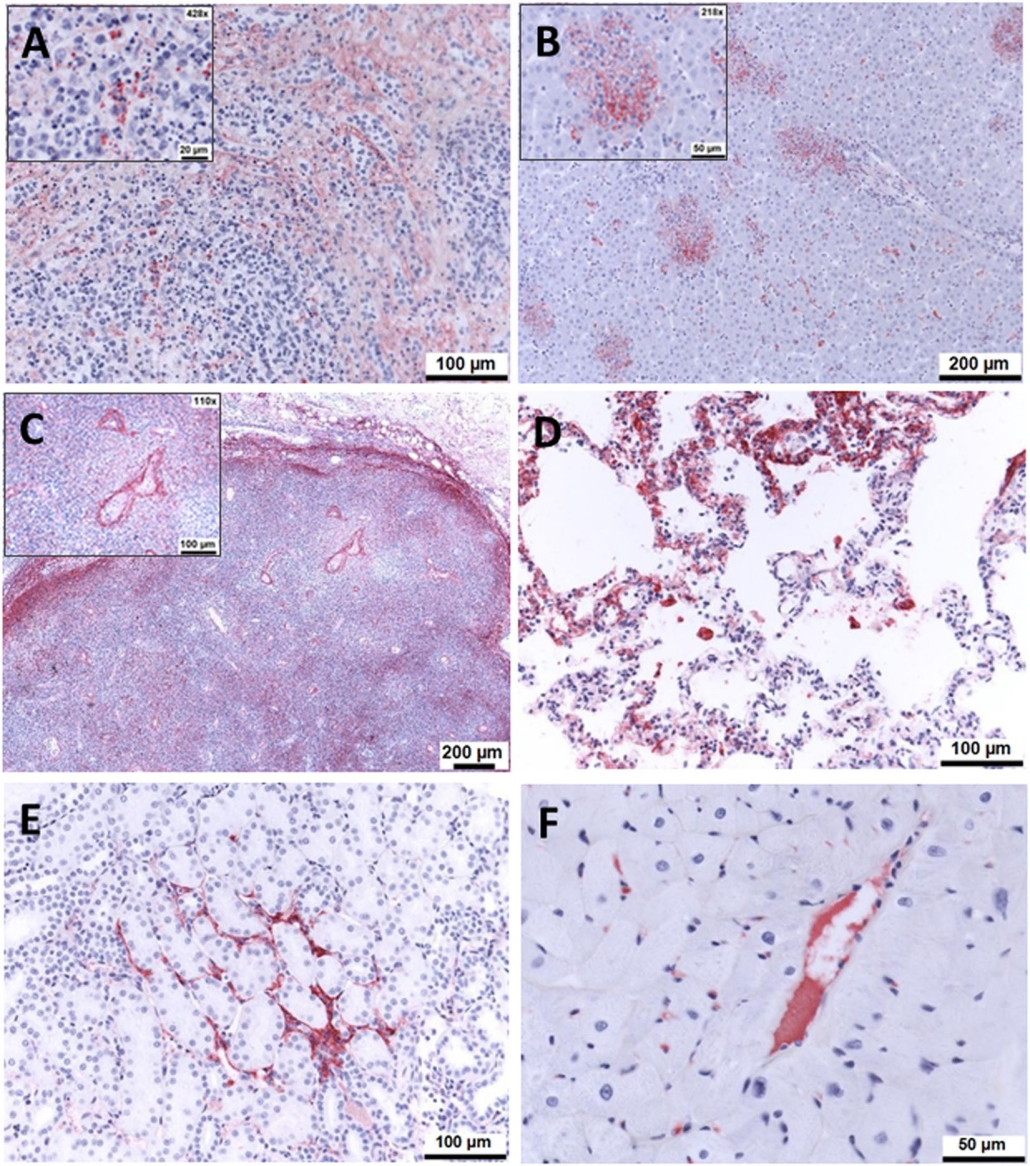
Immunohistochemistry staining of Ebola virus antigen in the tissue of EBOV-challenge animal treated with EBOTAb at 2 days post-challenge. Animal CCB081 met humane endpoints at day 12 post-challenge. (**A**) Spleen. A prominent number of cells are stained positively for EBOV antigen in the red and white pulp. Inset, higher magnification showing positively stained cells in the white pulp. (**B**) Liver. Strong, positive staining for viral antigen is present in necrotic areas. (**C**) Inguinal lymph nodes. Abundant staining of viral antigen is present, most prominently in the subcapsular space and within the endothelium of blood vessels. (**D**) Lung. Strong, patchy staining of viral antigen in alveolar walls and alveolar macrophages is present. (**E**) Kidney. Strong, focal staining located in connective tissue surrounding tubules. (**F**) Heart. Focal strong staining of small blood vessel and endomysium.

In spleen from untreated EBOV-challenge animals, numerous areas stained positive for viral antigen in the red pulp medullary sinuses and in the white pulp. Very few cells positive for virus antigen, with strong cytoplasmic staining, were observed scattered within the red pulp of animals treated with EBOTAb 1 day post-challenge. When EBOTAb treatment was delayed to 2 days post-challenge, one animal (CCB081) exhibited marked lesions in the red and white pulp, more prominent in the former, with large numbers of cells staining positive for EBOV antigen (Fig. 7A). Very few scattered, positively stained cells were noted in the red pulp of the two other animals in this group. Animals treated with EBOTAb on day 3 post-challenge, showed numerous cells staining positively within the medullary sinuses, as well as the white pulp in two animals (CCB085 and CCB130). Occasional, positively stained cells were noted in the red pulp of animal CCB079 whereas the remaining animal in this group showed no staining.

When liver samples were analysed, viral staining was observed in both untreated animals. When animals were treated with EBOTAb 1 day post-challenge, a single focus of necrosis was observed in animal CCA087l but this area stained negatively for viral antigen. However, positively stained cells were occasionally noted within portal triads in this animal. The remaining three animals were negative for viral antigen. When EBOTAb treatment was initiated 2 days post-challenge, in animal CCB081 there were positively stained cells seen frequently within lesions (Fig. 7B). These changes were absent from two animals (CCB037, CCB038), although viral antigen was present in occasional cells, often located near blood vessels, in animals CCB049. In the group treated with EBOTAb 3 days post-challenge, numerous areas stained positive in two of the animals whereas in the other two animals viral antigen was absent.

In the two untreated animals, cells in the inguinal lymph nodes stained positively for EBOV antigen. Very few cells were stained positively in the cortex and medulla of all four animals treated with EBOTAb 1 day post-challenge. When treatment was delayed to 2 days post-challenge, one of four animals showed no antigen staining whereas two animals, CCB081 and CCB049 showed abundant immunostaining (Fig. 7C) or smaller numbers of cells positive for viral antigen, respectively. When EBOTAb was delayed to 3 days post-challenge, staining was positive in three animals and negative in one animal.

During the analysis of lung samples from untreated animals, prominent viral antigen staining was present in both animals within alveolar walls and within some vessels. When treated with EBOTAb 1 day post-challenge, one animal demonstrated staining of individual cells in the peripheral connective tissues, with occasional vascular luminal contents stained diffusely red. There was variable staining of luminal contents in some vessels in the remaining three animals. In animals treated with EBOTAb 2 days post-challenge, pale staining of plasma within occasional vessels was present and strong staining for viral antigen was present in alveolar walls in animal CCB081 (Fig. 7D). In the group treated with EBOTAb 3 days post-challenge, patchy areas of viral antigen was stained in two of the animals and in the other two animals pale staining of plasma only was recorded.

Viral antigen was stained in the kidneys of untreated animals, multifocally within glomeruli, between tubules, and in peri-vascular and intra-vascular locations. IHC staining was variable between the groups treated with EBOTAb. In the group treated 1 day post-challenge, faint staining of plasma was observed and the remaining two animals were negative. In the 2 day post-challenge treated group, prominent staining was present in blood vessel lumina, focal areas of connective tissue between tubules, and occasionally within glomeruli, in one animal, CCB081 (Fig. 7E). Similar, but less prominent staining was present in animal CCB049 and no viral antigen was detected in animal CCB037. When EBOTAb treatment was delayed to 3 days post-challenge, positive staining was observed in two animals and the remaining two animals were negative.

Detection of viral antigen in heart was sporadic. In the untreated animals, only one animal of two tested was positive for staining in the blood vessels and endomysium; the remaining animals were negative. Similarly, in animals treated with EBOTAb 1 day post-challenge occasional, pale staining of plasma within blood vessels was observed in two animals (CCA087 and CCB053); whereas the remaining two animals were negative. In two of the three animals sampled that were treated with EBOTAb 2 days post-challenge, occasional, strong staining of some blood vessels and multifocal patches of endomysium was noted (Fig. 7F). When EBOTAb treatment was started 3 days post-challenge, positive staining of blood vessel and endomysium was observed in two animals whereas the remaining two animals were negative.

## Discussion

The results reported provide the first evidence demonstrating the protective efficacy of an ovine pAb therapy against EBOV in a NHP model. These findings are important as pAb confer several advantages to competing mAb-based therapies. Firstly, the production costs are low^39^. As EBOV mainly affects areas in the developing world, the cost of treatments is an important determinant of whether therapies are viable in lower middle income (LMI) countries. Secondly, the pAb contains a range of antibodies spanning a variety of epitopes, which reduces the risk of escape mutants arising as is possible using mAb-based therapies. This is demonstrated by a report when three neutralising mAb’s (comprising the treatment ZMAb) were tested in macaques against EBOV, resulting in one animal infected with EBOV containing two mutations in the EBOV glycoprotein which corresponded to the binding sites of the antibodies used^21^. Thirdly, pAb can be produced rapidly, with EBOTAb taking less than 6 months from concept to completion of efficacy testing in guinea pigs^28^. Fourthly, pAb-based products have a long history of successful usage in Africa, mainly with snake anti-venoms such as EchiTAb which has been used to treat >40,000 patients and is one of the most cost-effective therapies currently available^40^. Whilst equine species have been used for the production of pAb, equine immunoglobulin preparations suffer the disadvantage in that they contain high levels of IgG_T_, an antibody with the potential to cause allergic reactions^41^. When ovine and equine preparations were clinically compared against snake venom, the former induced mild reactions (pruritis) and the latter a severe reaction in some of the recipients (pruritus, urticaria, vomiting, cough, pyrexia)^42^; thus demonstrating the increased tolerability of ovine immunoglobulins. In addition, ovine Fab fragments have been used to treat cardiac glycoside toxicity, demonstrating a low incidence of adverse reactions and an absence of anaphylaxis^43^.

A challenge dose of 1000 pfu was used in this study since this dose, when given by various of routes of exposure, typically results in uniform lethality regardless of the EBOV strain^30^. This dose has been used by other studies with rhesus macaques^11, 12, 16, 20, 22, 24, 44–48^ and cynomolgus macaques^7, 8, 21, 23, 25, 36, 48–50^. Therefore, 1000 pfu is accepted as the suitable challenge dose to use in studies to assess filovirus countermeasures as agreed by an interagency and international group of scientists on the Filovirus Animal Non-Clinical Group (FANG)^51^. The intramuscular route of challenge is adopted by most laboratories conducting EBOV efficacy studies in NHPs and mimics a likely scenario of accidential needlestick injury^30^.

The challenge strain of virus used in the current studies was the Gabon 2001 strain, isolated from the first outbreak in the region associated with 92 cases and 72 deaths^52^. Subsequent outbreaks between Oct 2001-Dec 2003 accounted for 313 cases and 264 deaths in Gabon and the Republic of Congo^52^. When assessing countermeasures, successful interventions against a more aggressive strain can likely be adapted easily to less virulent strains^30^. Others have demonstrated differences in times to death between EBOV strains, including the recent 2014 Makona strain^49, 53^, 1995 Kitwit^11, 13, 14^ and the Mayinga strain isolated from the original outbreak in 1976^34, 37, 54^. However, despite the different strains used across EBOV NHP studies, the levels of lethality in untreated animals remain consistently high. The latest published information from FANG are that challenge stock should be low passage from a human case, not have been passaged in animals, are well characterised and of known genomic sequence^55^; all criteria which the Gabon 2001 used possess. However, in future it is likely that a single challenge strain is likely to be recommended between sites.

Haematology and blood biochemistry formed part of the study and very few differences were detected in the EBOTAb-treated groups compared with untreated animals. A similar observation was reported in experiments studying treatment with ZMapp, where treated NHPs survived infection, all animals presented with detectable abnormalities in blood cell counts and serum biochemistry during the course of the studies^24^. In biochemical analyses, the concentration of the liver enzymes ALT and AST were higher in the untreated group compared to the EBOTAb-treated group, indicative of liver dysfunction which is commonly associated with EBOV infection^56^. When blood cell counts were measured, a reduction in total lymphocytes was apparent in all groups before concentrations returned to starting values at the end of the study in surviving animals. A rapid loss of lymphocytes (CD4^+^, CD8^+^ and NK cells) has been reported as a feature of EBOV infection in cynomolgus macaques^36^. It should be noted that biochemical and haematological profiling was only conducted on a small number of timepoints in this study. This was adopted to ensure that animals only experienced the minimal of procedures and to preserve blood volume, particularly when studying a haemorrhagic fever virus disease. Whilst additional timepoints would undoubtedly have provided stronger data on the biochemistry and haematology parameters, as these were not the main purpose of the study blood sampling from all animals was kept to a minimum.

Although this study has demonstrated a protective efficacy for EBOTAb when treatment was delayed up to day 3 post-challenge (25% survival), other reports have demonstrated complete survival in macaques following administration of a single^45^ or multiple mAbs^24^ at 5 days post-challenge or a small-molecule antiviral compound at 3 days post-challenge^47^. However, both of these studies utilised the rhesus macaque model. The length of disease before death or reaching humane end points is generally longer by one to two days in rhesus macaques^48^ and reports demonstrating positive effects of post-exposure treatments against EBOV have predominantly used this macaque species^10–16, 47^. The EBOTAb results reported here, and those of other studies that used cynomolgus macaques^21^, which have a shorter disease course, may have demonstrated improved survival rates if tested in rhesus macaques^13^. The NHP model for EBOV used in this report represents a worst-case scenario, as it results in 100% of animals reaching humane endpoints. Data from the 1976 Ebola disease outbreak in the former Zaire was 100% in 85 cases associated with injection, versus approximately 80% in 149 cases of documented exposure^1^. This supports the view that the therapeutic window in natural EBOV infection would be longer than that modelled in NHP studies^12^.

Blood was collected from all animals 7 days post-EBOV challenge. This time-point was chosen due to trying to capture the peak time of viremia in untreated animals from previous experience and that of others^49^; however, one animal met humane endpoints prior to this time. Given the outbred nature of cynomolgus macaques, individual variation in peak viremia response would be expected, but a single timepoint was scheduled to reduce blood withdrawal procedures. No PCR signals were detected in the blood at this timepoint in animals treated with EBOTAb on day 1 or day 2; although 3 of 4 animals treated at day 3 did have EBOV-specific RNA present. No PCR signals or viable virus counts were detected at the scheduled endpoint, day 14 post-challenge in EBOTAb-treated animals, which survived a lethal EBOV challenge. Normally, the levels of both parameters are measured as sometimes significant viral RT-PCR signals are detectable whilst live viral assays are negative^20^. In our study, both RT-PCR and plaque assay gave similar kinetics of responses. Failure to detect virus in the surviving EBOTAb-treated macaques gives credence to the therapy clearing the EBOV challenge. Whilst not tested in this study, others have demonstrated that in NHPs protected from lethal EBOV challenge following antibody treatment have sustained protection against re-exposure to EBOV^23^.

Whilst EBOTAb has previously been shown to exert strong neutralisation activity against EBOV^28^, in animals protected from a vesicular stomatitis virus (VSV)-based vaccine post-exposure it was the rapid development of non-neutralising antibody that was important and neutralising antibodies were not essential for infection control^13^. However, due to the mechanism of action of the VSV vaccine, the protection is likely to be multifactorial. Likewise, further study is required to identify the components and mechanisms of action of EBOTAb, which are also likely to be multidisciplinary due to the range of the binding epitopes on the EBOV glycoprotein.

For the study, macaques were administered with EBOTAb on five consecutive days followed by a further three treatments every other day. This daily dosing during the perceived critical stages of EBOV disease was based on the half-life of other ovine antibody preparations in humans being between 4 hours^57, 58^ to 20 hours^59, 60^. Having shown that EBOTAb confers postexposure protection against EBOV, to refine the dosing schedules pharmacokinetic studies with EBOTAb are warranted. A similar preparation developed by the same manufacturer against Clostridium difficle (PolyCAb) is undergoing a Phase I clinical trial^61^; thus, data from this trial will be valuable to apply to EBOTAb. The dosing concentration used in this study was 340.8 mg EBOTAb per animals, so based on the starting weights of the animals ranging from 2.78–3.33 kg the concentration was 102.3–122.5 mg/kg. Previously, the specificity of EBOV-specific antibody in EBOTAb was shown to be 10.2%^28^ so the concentration of EBOV-specific antibodies would be 10–12 mg/kg. Concentrations of monoclonal antibodies tested in non-human primate EBOV studies have varied from 16.7 mg/kg^20^ to 50 mg/kg^24^. Thus, the levels of EBOV-specific antibodies are at the lower end of this spectrum.

To treat EBOV infection, it is likely that multiple therapies may be considered either solely or in combination. For antibody treatment, adenovirus-vectored human interferon (IFN)-α^62^ has been demonstrated to extend the treatment window in NHPs^63^. Due to the complexities of testing multiple therapies and the ethical and financial considerations of primate usage for scientific research, testing combination therapies is problematic and therapies are usually only tested on their own. However, this does not preclude the possibility of supplementing treatment with EBOTAb with another treatment option, e.g. favipiravir^64^, that is being assessed in the NHP model of EBOV and is heat-stable and cost-effective like EBOTAb, so is of value to use in LMI countries.

In summary, the results reported here demonstrated that EBOTAb protected cynomolgus macaques from EBOV infection, even demonstrating a level of efficacy when given as late as 3 days post-challenge. This data supports the results reported showing efficacy of EBOTAb in the guinea pig model of EBOV disease^27, 28, 55^. Clearly pAb treatment of EBOV has some advantages over mAb-based therapies. Our data suggest that EBOTAb is as effective as mAb treatment for EBOV and warrants clinical development. It is our objective to complete additional preclinical studies (e.g. pharmacokinetic studies, dose escalation and toxicity studies) prior to advancing into safety studies of EBOTAb in a phase I clinical trial so when the next EBOV outbreak occurs it can be rapidly deployed and its therapeutic potential assessed in humans.

## Methods

### Ethics statement

Work on NHPs was performed in the INSERM Jean Mérieux BSL-4 laboratory, Lyon, France (French Animal regulation comitée N° B69 387 05 02). NHPs were housed and manipulated according to the guidelines of Directive 2010/63/UE. Animals were handled in strict accordance with good animal practice as defined by the French national charter on the ethics of animal experimentation. Approval from the ethical committee (common ethical committee for 10 Laboratory, recorded at the Ministry for Higher education and Research with the number CE015) for this study was identified by CECCAPP_P4_2015_004 and the project was carried out in accordance with this authorisation. Furthermore, each experiment was conducted out by experienced staff and in interaction with a veterinary surgeon.

### Animals

Fifteen, young male adult (27 months) cynomolgus macaques (*Macaca fascicularis*) weighing 2–4 kg were sourced from a conventional breeding colony (Silabe, Strasbourg, France). Four animals were assigned to each of three treatment groups and 3 to the control group. Group sizes were kept to a minimum whilst still conferring statistical significance between treated and the untreated control group. Tap water was available at 730 ml/day and animals fed with 100 g/day of old world monkey mix (supplied by SDS). The animals’ health and welfare was monitored on a daily basis. In accordance with BSL-4 procedures; animals were housed individually. Pen sizes and internal environment controls met the requirements of EU legislation. Environmental enrichment in animal enclosures was adapted to the individual needs of NHPs. To prevent any suffering to animals, humane endpoints were identified: anorexia and dehydration over four days; epistaxis; haemorrhagic diarrhoea over two days; mucous membrane diarrhoea over two days; pirexia higher than 41 °C over two days (in combination with anorexia); temperature lower than 35 °C; installation of a state of prostration (in combination with anorexia); weight loss of 15% (compared with Day 0); no awakening 120 minutes after anaesthesia. Once animals met a humane endpoint, they were immediately euthanised.

### EBOV challenge

Animals were challenged with strain Gabon 2001 of EBOV from the Centre for Medical Research in Franceville (CIRMF), originally isolated from a lethal case. Virus was diluted in sterile PBS to contain 1000 pfu within a volume of 0.5 ml, which was then delivered via the intramuscular route to anesthetised animals. All animals wer challenged at the same time, with 140 minutes between the first animal and the last animal. Challenge virus was backtitrated to confirm dose was within expected levels.

### EBOTAb preparation

EBOTAb was produced as previously described^28^. Briefly, three sheep were immunised subcutaneously into six sites on each sheep (neck and groin) with 0.5 mg mammalian-expressed recombinant EBOV glycoprotein mixed with adjuvant (Freund’s complete for primary immunisation and Freund’s incomplete for secondary immunisations). Specific antibody titres were determined by ELISA and when a plateau of immune response was reached (approximately 14 weeks after the first immunisation), 10 ml of blood per kg body weight was collected every four weeks. The IgG fraction was extracted from the ovine sera by the addition of caprylic acid (octanoic acid) followed by dilution with saline and mixed vigourously to precipitate non-IgG proteins^65^. The EBOTAb product was formulated in 20 mM citrate buffer (pH 6.0 ± 0.2) containing 153 nM NaCl and 0.1% Tween 20, pH 6.0. Purity was assessed by size-exclusion gel filtration chromatography.

### EBOTAb administration

On treatment days, animals were given 6 ml of EBOTAb (final concentration 56.8 g/L) by infusion over six minutes via the intravenous route. Experimental treatment was on 5 consecutive days followed by three further treatments every other day. The difference between treatment groups was the starting day for EBOTAb treatment: day 1, 2 or 3 post-challenge.

### Sample preparation

Blood was collected from animals into two dry tubes of 2 ml and one EDTA tube of 3 ml tubes, which were spun at 4700 rpm during 10 minutes before plasma and serum were aspirated off and stored at −80 °C. At necropsy, sections of liver and spleen were collected into dry tubes and stored at −80 °C in dry tubes. To prepare samples for PCR analysis, 100 µL of samples (plasma or supernatant of crushed organs) were inactivated by addition to 400 µL of AVL (Qiagen buffer). After 10 minutes at room temperature, 400 µL of ethanol were added. In addition to liver and spleen, samples from inguinal lymph nodes, kidney, lung and heart were placed into 10% formalin solution for histological analysis.

### RT-PCR

RNA from plasma and tissue homogenates prepared from liver and spleen was isolated using a Qiagen extraction kit (QIAamp Viral RNA mini kit). RT-PCR were performed on eluates of extraction. The EBOV RT-PCR assay was then conducted by using the Bio-Rad CFX96 real-time PCR system (Bio-Rad Laboratories, Hercules, CA, USA; P/N 172–5200) allowing amplification of the NP gene. The primers and probes, which were used in these 2 assays for targeting the NP genes of EBOV were detailed below:

Amplicon of 485 bp:

enpT-F: TAATACGACTCACTATAGGGATGCCGGAAGAGGAGACAA

enpT-R:TAATACGACTCACTATAGGGCGGGCGAAAGGAGCATA

Amplicon of 80 bp:

enp-F: GCAGAGCAAGGACTGATTCA

enp-R:GTTCGCATCAAACGGAAAAT

enp-Probe: FAM-CAACAGCTTGGCAATCAGTTGGACA-TAMRA

Prior to amplification the RNA was reverse transcribed at 50 °C for 30 min. This was followed by one cycle of denaturation at 95 °C for 15 min. Next, RT-PCR amplification was carried out for 45 cycles at 94 °C for 15 s and 59 °C for 30 s. The fluorescence was read at the end of this second step allowing a continuous monitoring of the amount of RNA. Quantification was based on a viral RNA standard using the BIO-RAD software.

### Plaque assay

Viable viral loads in liver and spleen homogenates were measured by plaque assay. Standard 12-well microplates of Vero E6 cells (supplied from the European Collection of Cell Cultures, ECACC, UK) were prepared the day before titration. Cells were infected with dilutions of samples during 1 hour at 37 °C. After incubation, Dulbecco minimal essential medium supplemented with 2% fetal bovine serum and 1% penicillin/streptomycin was added and incubated at 37 °C for 7 days. Titer was determined by immunostaining with a specific antibody.

### Biochemistry

Enzymes (ALP, ALT, AST), substrates (bilirubin, creatine, urea) and specific protein (CRP) were obtained using a Pentra C200 Analyser (Horiba, Kyoto, Japan).

### Haematology

Total leukocyte counts; lymphocyte, platelet, erythrocyte counts, hemoglobin levels, and hematocrit values were determined from blood containing EDTA by using the MS9–5s Hematology Analyser (Melet Schloesing, Osny, France).

### Histology and IHC

Tissue samples that had been fixed in 10% formalin solution for at least 21 days were processed routinely to paraffin wax. Sections were cut at 3–5 μm, stained with haematoxylin and eosin (HE) and examined microscopically. For immunohistochemistry, sections were stained for EBOV antigen using the Leica BondMax (Leica Biosystems) and the Leica Bond Polymer Refine Detection kit (Leica Biosystems). An antigen retrieval step was included for 10 minutes using the Bond Enzyme Pretreatment kit, enzyme 3 (3 drops). A rabbit polyclonal, anti-EBOV VP40 antibody (IBT Bioservices) (dilution 1:2000) was incubated with the slides for 60 minutes. DAB chromogen and haematoxylin counterstains were used to visualise the slides.

### Statistical analysis

Data was analysed with Minitab (version 16.2.2) to determine statistical significance. Due to the small group sizes and data not being proven to be normally-distributed, a non-parametric Mann-Whitney statistical test was used. Assessing the differences between groups for biochemical and haematological markers, the lowest limit was P = 0.0518 so this was used as the level to infer significance.
